# Microfabrication Process-Driven Design, FEM Analysis and System Modeling of 3-DoF Drive Mode and 2-DoF Sense Mode Thermally Stable Non-Resonant MEMS Gyroscope

**DOI:** 10.3390/mi11090862

**Published:** 2020-09-17

**Authors:** Syed Ali Raza Bukhari, Muhammad Mubasher Saleem, Umar Shahbaz Khan, Amir Hamza, Javaid Iqbal, Rana Iqtidar Shakoor

**Affiliations:** 1 Department of Mechatronics Engineering, National University of Sciences and Technology, Islamabad 44000, Pakistan; saraza.mts19ceme@mts.ceme.edu.pk (S.A.R.B.); u.shahbaz@ceme.nust.edu.pk (U.S.K.); a.hamza@ceme.nust.edu.pk (A.H.); j.iqbal@ceme.nust.edu.pk (J.I.); 2 National Centre of Robotics and Automation(NCRA), Islamabad 44000, Pakistan; rana.iqtidar@mail.au.edu.pk; 3 Department of Mechatronics Engineering, Air University, Islamabad 44000, Pakistan

**Keywords:** MEMS gyroscope, multi-degree of freedom (multi-DoF), non-resonant, microfabrication, thermal stability, finite element method (FEM), system modeling, robustness

## Abstract

This paper presents microfabrication process-driven design of a multi-degree of freedom (multi-DoF) non-resonant electrostatic microelectromechanical systems (MEMS) gyroscope by considering the design constraints of commercially available low-cost and widely-used silicon-on-insulator multi-user MEMS processes (SOIMUMPs), with silicon as a structural material. The proposed design consists of a 3-DoF drive mode oscillator with the concept of addition of a collider mass which transmits energy from the drive mass to the passive sense mass. In the sense direction, 2-DoF sense mode oscillator is used to achieve dynamically-amplified displacement in the sense mass. A detailed analytical model for the dynamic response of MEMS gyroscope is presented and performance characteristics are validated through finite element method (FEM)-based simulations. The effect of operating air pressure and temperature variations on the air damping and resulting dynamic response is analyzed. The thermal stability of the design and corresponding effect on the mechanical and capacitive sensitivity, for an operating temperature range of −40 °C to 100 °C, is presented. The results showed that the proposed design is thermally stable, robust to environmental variations, and process tolerances with a wide operational bandwidth and high sensitivity. Moreover, a system-level model of the proposed gyroscope and its integration with the sensor electronics is presented to estimate the voltage sensitivity under the constraints of the readout electronic circuit.

## 1. Introduction

The gyroscopes detect rotation of an object and are an integral part of Inertial Measurement Units (IMUs) which enable the development of self-contained and high precision Inertial Navigation Systems (INSs). In comparison to traditional mechanical gyroscopes with rotating parts, ring-laser and fibre-optic gyroscopes, the microelectromechanical systems (MEMS) gyroscopes offer small size, are lightweight and low-cost owing to batch fabrication, low power consumption and integration with integrated circuit (IC) technology.

The working of vibratory MEMS gyroscopes is dependent on the Coriolis effect which requires oscillations of the proof mass to be sustained in the drive axis and a sensing mechanism for the measurement of displacement of the proof mass corresponding to an input rotation. In MEMS gyroscopes, the oscillations in the proof mass and sensing of its displacement are achieved by using different transduction mechanisms including electrostatic [1], electrothermal [2], piezoelectric [3], piezoresistive [4] and optical [5]. Among these transduction mechanisms, the electrostatic is most commonly used due its fast response time, low power consumption¸ ease of fabrication and integration with the drive and readout electronics [6].

Based on the working principle, the MEMS gyroscopes are divided into two main categories including resonant and non-resonant gyroscopes. In resonant MEMS gyroscopes, the device is operated at resonance and both the drive and sense mode resonant frequency values are generally matched which leads to high mechanical sensitivity [7]. The reliability of resonant MEMS gyroscopes is of major concern since a mismatch between the drive and sense mode resonant frequencies occurs due to microfabrication process tolerances and fluctuations in the operating temperature and pressure conditions [8,9]. Thus, resonant MEMS gyroscope requires strict frequency control using feedback electronics [10] or electrostatic frequency tuning [11].

The non-resonant MEMS gyroscopes make a multi-degree of freedom (multi-DoF) system with masses coupled to each other through mechanical suspension in either the drive or sense mode. The non-resonant MEMS gyroscope works in the flat region between the resonant peaks of a multi-DoF system and offers inherent robustness against fabrication process and environmental variations. The main challenges in the design of multi-DoF MEMS gyroscope involve increasing both mechanical sensitivity and operational bandwidth. The earliest design approach, based on the two-mass system in the drive mode utilizing the dynamic vibration absorber concept, was presented in [12]. This approach was used and built upon by different researchers for the design of non-resonant MEMS gyroscopes [13,14,15]. The main drawback of this two-mass system-based approach is that the operational bandwidth and mechanical gain of the MEMS gyroscope can only be increased by optimizing the mass and resonant frequency ratio of masses that are coupled together. To overcome this issue, a modified vibration absorber design concept has been proposed in [16] with the addition of a grounded mechanical spring that is attached to the absorber mass. The proposed design concept overcomes the requirement of having very small absorber mass but does not resolve the issue of variation in the stiffness of mechanical springs due to fabrication imperfections or high operating temperature values. A multi-mass-based system with the addition of collider mass between the drive and sense mass has been presented in [17] to minimize the effect of operating conditions and to achieve higher dynamic amplification. The collider mass in this design approach transmits the dynamic energy from the drive mass to the final oscillating sense mass. Previously, the authors have presented the implementation of the collider mass concept, for the transfer of energy from drive mass to sense mass, for a 3-DoF drive and 1-DoF sense mode electrothermally actuated MEMS gyroscope [18]. The MEMS gyroscope was optimized considering the microfabrication process constraints of MetalMUMPs process with 20 μm thick structural layer of nickel. In comparison to our previous work, in this paper, we present an electrostatic multi-DoF MEMS gyroscope, based on the collider mass concept presented in [17], with 3-DoF drive and 2-DoF sense mode. The proposed design is optimized using commercially available SOIMUMPs microfabrication process with 25 μm thick structural layer of silicon. The 2-DoF sense mode and the fact that the silicon is more thermally stable in comparison to nickel, allows to achieve higher robustness, in the operating temperature range of −40 °C to 100 °C, for the proposed MEMS gyroscope. Table 1 summarizes the multi-DoF-based non-resonant MEMS gyroscopes presented in the literature along with their material, structural layer thickness, device size, sensitivity and operational bandwidth. The sensitivity values vary in units owing to the fact that in the literature either mechanical, capacitive or voltage sensitivity values are reported for a respective MEMS gyroscope design. Most of these designs utilize a multi-DoF design concept in either the drive or sense mode, but not in both.

The operation of a MEMS gyroscope is strongly dependent on the operating temperature [24]. The high-temperature values may lead to stiffness degradation of the mechanical springs, variations in the air damping force and misalignment of the sensing parallel plates in electrostatic MEMS devices due to thermal expansion. Generally, for the MEMS gyroscopes, different temperature compensation techniques are implemented after the fabrication for reliable operation [25,26], which results in added complexity. Thus, both the selection of thermally stable material for the fabrication of MEMS gyroscope and structural design optimization considering the temperature effects must be considered at the design level.

The electrical sensitivity of a MEMS gyroscope is not only dependent on the structural design, but also on the readout electronics. For the MEMS gyroscopes, that work on the electrostatic actuation and sensing principle, it becomes very important to analyze the device performance in integration with the sensing electronics at the design phase. Most of the works presented in the literature on the MEMS gyroscope design generally lack this aspect. In this paper, we present the design of a multi-DoF non-resonant electrostatic MEMS gyroscope with 3-DoF drive and 2-DoF sense mode oscillators to achieve both wide operational bandwidth and high mechanical sensitivity. The design is optimized considering the commercially-available SOIMUMPs fabrication process with a structural layer of 25 µm thick silicon material. The effect of temperature and pressure variations on the air damping and dynamic behavior of the MEMS gyroscope is analyzed in detail. Moreover, the integration of the proposed MEMS gyroscope with readout electronics is discussed in detail.

## 2. Structural Design of Multi-Degree of Freedom (Multi-DoF) Microelectromechanical Systems (MEMS) Gyroscope

**Figure 1:**
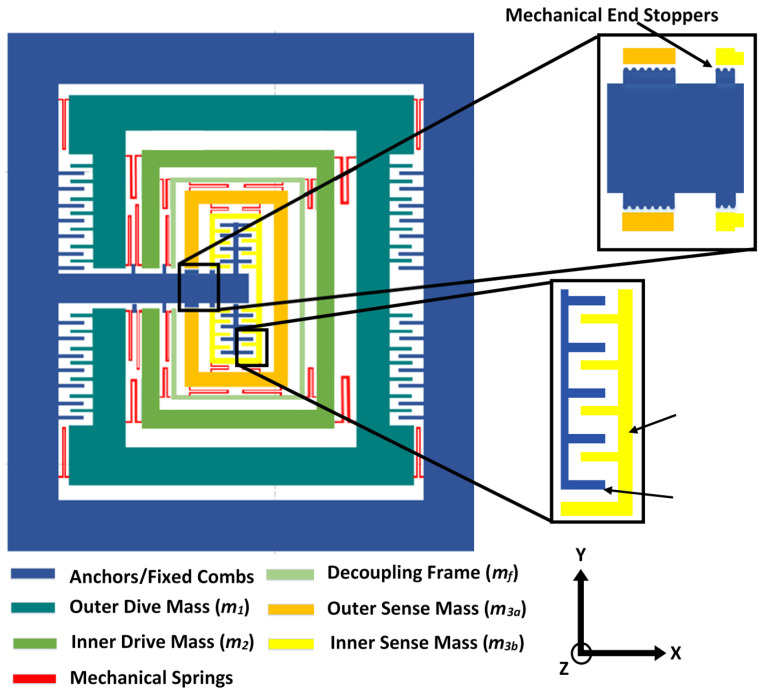
Proposed multi-degree of freedom (multi-DoF) microelectromechanical systems (MEMS) gyroscope design with 3-DoF drive mode and 2-DoF sense mode oscillators.

Figure 1 shows the proposed multi-DoF MEMS gyroscope design with a 3-DoF drive mode and 2-DoF sense mode oscillators. The drive and sense masses are connected to each other using serpentine-shaped mechanical springs. These springs allow the displacement of the masses in the desired direction only, thus minimizing the cross-axis displacement. The oscillations in the drive axis (*x*-axis) are obtained through a comb drive-based electrostatic actuator which is attached to the outer mass $m_{1}$. The combination of mass $m_{3a}$ and $m_{3b}$ along with the decoupling frame of mass $m_{f}$, acts as a final vibration absorber $m_{3}$. For an applied actuation voltage to the electrostatic actuator, a dynamically-amplified displacement is achieved in the mass $m_{3}$ while the mass $m_{2}$ transfers the dynamic energy from mass $m_{1}$ to $m_{3}$. For an input rotation in the *z*-axis, a Coriolis force is generated in the orthogonal axis to drive and rotation axes i.e., *y*-axis. This leads to a displacement in the 2-DoF sense mode oscillator consisting of a mass $m_{3a}$ and $m_{3b}$, which are connected to each other through mechanical springs. The mass $m_{3b}$ acts as a vibration absorber for the mass $m_{3a}$ in the sense direction, resulting in a dynamically-amplified displacement in the mass $m_{3b}$ with respect to mass $m_{3a}$ corresponding to the Coriolis force. The displacement in the mass $m_{3b}$ is sensed using parallel plates, which are arranged in a gap–antigap-based differential configuration [27]. The small gap ($d_{1}$) is kept as 3 µm while the large gap ($d_{2}$) value is 9 µm. The change in capacitance in the sensing parallel plates has an inverse relationship to the initial air gap between the plates. The minimum air gap allowed by the microfabrication process is 2 µm. However, to meet the microfabrication process constraints and to minimize the Brownian noise and air damping effect, the initial small gap ($d_{1}$) is considered to be 3 µm. To minimize the effect of sudden mechanical shock, semicircle-shaped end stoppers are designed at the end of the masses adjacent to the anchors. Table 2 shows the main design parameters of the multi-DoF MEMS gyroscope.

## 3. Microfabrication Process

**Figure 2:**
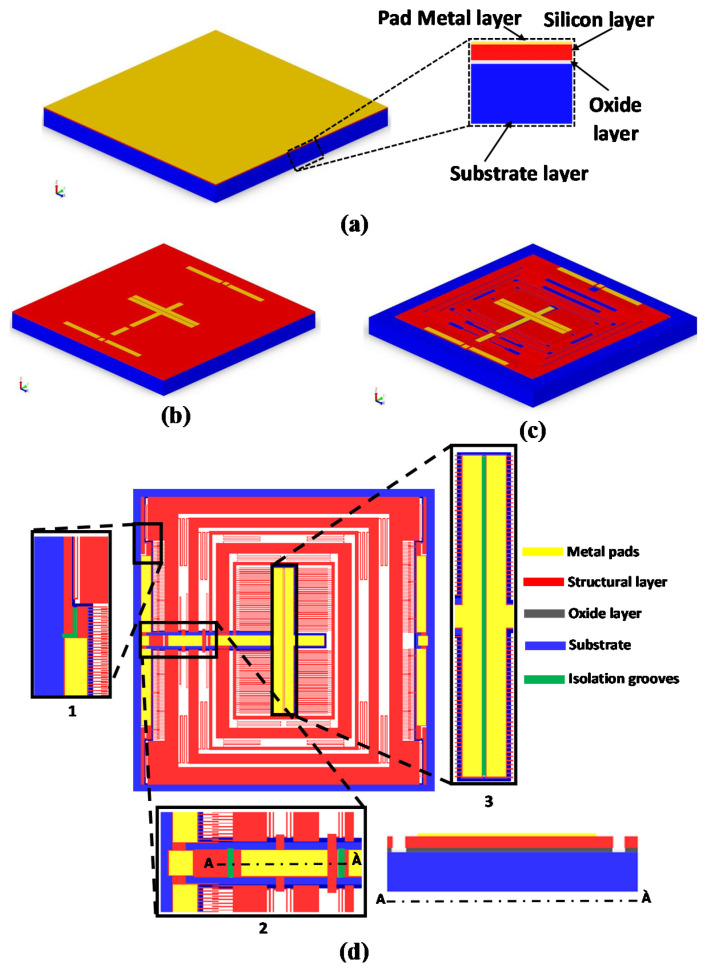
Microfabrication process steps for the proposed MEMS gyroscope design.

The MEMS gyroscope is designed following the constraints of the commercially available and low-cost multiuser SOIMUMPs micromachining process, offered by MEMSCAP Inc. USA [28]. In addition to being a mature fabrication process, SOIMUMPs offers to fabricate high aspect ratio microstructures with minimum air damping, due to absence of substrate below the moving microstructure. Both of these attributes are generally desired in the design of high-performance MEMS inertial sensors. The SOIMUMPs microfabrication process is a single-wafer silicon-on-insulator (SOI) micromachining process and allows patterning and etching on the SOI wafer using four mask layers with different thickness. The topmost layer, called Padmetal, is followed by a device layer of 25 µm thickness. An oxide layer of 2 µm thickness is sandwiched between the device layer and the 400 µm thick substrate layer. The minimum feature size for the device layer is 2 µm, but to avoid buckling due to the residual stresses it is suggested, by the foundry, that the beams must have a thickness of 6 µm or more for a length of more than 100 µm. Figure 2 shows the details of the microfabrication process step for the proposed MEMS gyroscope design. The main steps involved are;

(a) The process starts with an SOI wafer as shown in Figure 2a. The doping of the top layer of silicon is done by depositing a phosphosilicate glass (PSG) layer and then annealing is done at 1050 °C in Argon. It is followed by the removal of the PSG layer using wet etching.
(b) The lift-off process is then used to deposit the first Padmetal layer which consists of 500 nm gold stack and 20 nm chrome layer, as shown in Figure 2b.
(c) To apply a second mask, a lithography technique is utilized for the patterning of the silicon layer as shown in Figure 2c.
(d) To apply the TRENCH mask, substrate layer of thickness 400 µm is patterned in which lithography technique is used with successive etching through Deep Reactive Ion Etching (DRIE). The final structure is obtained by removing the buried oxide layer in the regions defined by the TRENCH mask, as shown in Figure 2d.

The electrical isolation between the top structural layer of silicon and substrate is achieved using an Oxide layer (silicon oxide layer). However, since there is no insulating layer between the top PadMetal layer and structural silicon layer, so the PadMetal layer patterned on different parts of the structure is electrically connected to each other due to structural doping of the silicon. To achieve the electrical isolation between the fixed and moving combs in the electrostatic actuator and parallel displacement sensing plates, 5 μm wide and 25 μm deep isolation grooves, of different lengths, are made in the structural layer of silicon as shown in Figure 2d. The zoomed sections 1, 2 and 3 show the location of the various isolation grooves. The grooves shown in zoomed section 1, disconnect the movable masses from the fixed combs and are repeated on all four corners of the MEMS gyroscope. The zoomed section 2 shows two isolation grooves which isolate the oscillating masses from the fixed actuator combs and sensing parallel plates. The zoomed section 3 shows the isolation groove between the two sets of fixed sensing parallel plates for the electrical isolation.

### 3.1. Microfabrication Process Limitations

For the accurate release of microstructures and structural integrity, the SOIMUMPs microfabrication process has certain design rules to be followed. For the complex MEMS devices, like MEMS gyroscopes, this limits the design options for the MEMS designer. The main limitations that are considered for the proposed multi-DoF MEMS gyroscope design are discussed below.

#### 3.1.1. Limitation 1

**Figure 3:**
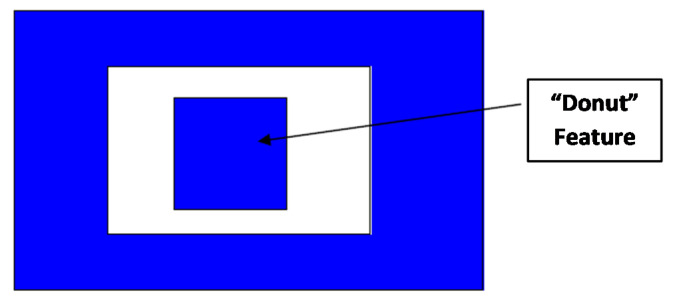
Schematic of invalid doughnut/anchor feature in the SOIMUMPs process.

The design cannot have a central anchor as shown in Figure 3. This is because such an anchor/doughnut feature does not survive during the microfabrication etching step and eventually falls out. For the proposed multi-DoF MEMS gyroscope, this implies that in comparison to traditional multi-DoF MEMS gyroscope designs reported in the literature, the sense mass cannot be completely enclosed within another mass due to the requirement of anchor part for the fixed parallel sensing plates. Thus, for the proposed MEMS gyroscope, the anchor for the fixed parallel sensing plates attached to mass *m*_3*b*,_ is provided by routing the silicon fixed beams from one side of the outer mass *m*_1_ as shown in Figure 1.

#### 3.1.2. Limitation 2

As the silicon substrate is completely etched in the SOIMUMPs process, bottom electrodes for the out-of-plane actuation and sensing cannot be fabricated in this process. This leaves the MEMS designer to use only in-plane actuation and sensing techniques.

#### 3.1.3. Limitation 3

The silicon layer is the structural layer in which the device is patterned, as shown in red in Figure 2. As per the design rules of the SOIMUMPs process, the etched part of the silicon layer must be less than 33% of the total chip area. This limitation is imposed to make sure that the structural integrity of the device remains intact after fabrication. For the proposed MEMS gyroscope, this rule limits the amount of allowed spacing between the masses and hence on the number of turns of mechanical springs.

### 3.2. Process Driven Design Modifications

Some design choices had to be made in order to mitigate the limitations imposed by the microfabrication process. The first limitation states that an anchor in the middle surrounded by empty space is not permitted. To remedy this, the fixed parallel sensing plates are partially enclosed within the sense mass $m_{3b}$. Ideally, the anchor should have been completely enclosed to make the MEMS gyroscope design symmetric. However, this is not permitted, so all the masses have to be altered so that support can be provided to the central anchor from the external anchor. This approach allows to alter the design minimally and keep the design simple and as symmetric as possible. Although the device does not remain completely symmetric after this modification, the effects of this asymmetry on the performance of the device are negligible. This is verified through finite element method (FEM)-based thermal stability analysis, discussed in Section 5.5 and Section 5.6. The second limitation is the absence of a bottom electrode. Thus, for the proposed MEMS gyroscope, we have used in-plane comb drive actuators and in-plane parallel plate-based displacement sensing mechanism. The third limitation is to keep the etched area in the structural layer less than 33% of the total device area. This is achieved by minimizing the air gaps between the masses and by reducing the unused empty spaces in the design.

## 4. Analytical Modeling of the Multi-DoF MEMS Gyroscope

### 4.1. Equations of Motion for the Drive and Sense Mode Oscillators

**Figure 4:**
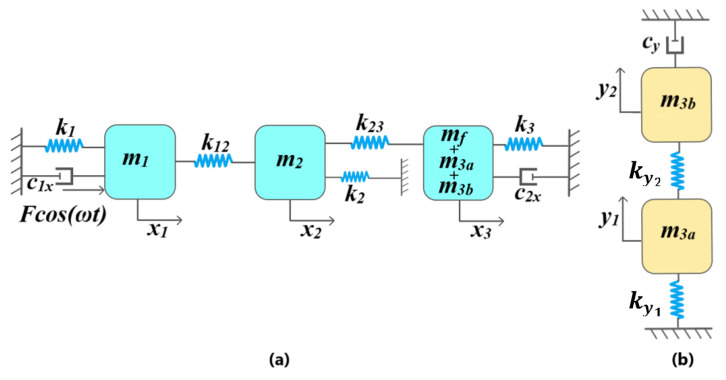
Discrete mass–spring–damper model for the proposed MEMS gyroscope. (**a**) Drive mode and (**b**) sense mode.

Figure 4a shows the 3-DoF drive mode mass–spring–damper model representation of the proposed MEMS gyroscope. For an input driving force Fcos(ωt), displacement of mass $m_{1}$, $m_{2}$ and sense mass ($m_{3a}$, $m_{3b}$, $m_{f}$) is represented by $x_{1}$, $x_{2}$, and $x_{3}$ respectively. The mass $m_{1}$ and sense mass ($m_{3a}$, $m_{3b}$, $m_{f}$) are anchored to the substrate through mechanical springs $k_{1}$ and $k_{3}$ respectively. The mass $m_{2}$, attached to the mass $m_{1}$ and sense mass ($m_{3a}$, $m_{3b}$, $m_{f}$) through mechanical springs $k_{12}$ and $k_{23}$, respectively, acts as a collider mass and transfers the dynamic energy from the mass $m_{1}$ to the sense mass ($m_{3a}$, $m_{3b}$, $m_{f}$). The mass $m_{2}$ is also connected to the substrate through mechanical spring $k_{2}$. The $k_{2}$ allows us to optimize the dynamic displacement amplification between the mass $m_{1}$ and sense mass ($m_{3a}$, $m_{3b}$, $m_{f}$) and the width of the flat operational region between the resonance peaks of drive mode oscillators [17]. The air damping between the actuator combs attached to the mass $m_{1}$ and sensing parallel plates attached to sense mass ($m_{3a}$, $m_{3b}$, $m_{f}$) are represented by $c_{1x}$ and $c_{2x}$, respectively. Figure 4b shows the 2-DoF sense mode mass–spring–damper model of the MEMS gyroscope. The mass $m_{3b}$ acts as dynamic vibration absorber for the mass $m_{3a}$ and both masses are coupled through the mechanical spring $k_{y_{1}}$. The mass $m_{3a}$ is attached to the decoupling frame $m_{f}$, which acts as an anchor, through mechanical spring $k_{y_{2}}$ and the air damping in the sensing parallel plates attached to the mass $m_{3b}$ is represented by $c_{y}$.

For an angular rate of Ω in the z-axis, the second-order differential equations of motion for the proposed MEMS gyroscope written as:

(1) $$m_{1}{\ddot{x}}_{1}+c_{1x}{\dot{x}}_{1}+k_{1}x_{1}+k_{12}\left(x_{1}-x_{2}\right)=F_{D}$$

(2) $$m_{2}{\ddot{x}}_{2}+k_{2}x_{2}+k_{12}\left(x_{2}-x_{1}\right)+k_{23}\left(x_{2}-x_{3}\right)=0$$

(3) $$\left(m_{f}+m_{3a}+m_{3b}\right){\ddot{x}}_{3}+c_{2x}{\dot{x}}_{3}+k_{3}x_{3}+k_{23}\left(x_{3}-x_{2}\right)=0$$

(4) $$m_{3a}{\ddot{y}}_{1}+k_{y_{1}}y_{1}+k_{y_{2}}\left(y_{1}-y_{2}\right)=F_{c_{(}m_{3a)}}$$

(5) $$m_{3b}{\ddot{y}}_{2}+c_{y}{\dot{y}}_{2}+k_{y_{2}}\left(y_{2}-y_{1}\right)=F_{c_{(}m_{3b)}},$$

where $F_{D}=Fcos\left(\omega t\right)$ is the driving actuation force, $F_{c_{(}m_{3a)}}=-2m_{3a}\Omega {\dot{x}}_{3}$ and $F_{c_{(}m_{3b)}}=-2m_{3b}\Omega {\dot{x}}_{3}$ are the Coriolis forces acting on the 2-DoF sense mode oscillator.

### 4.2. Calculation of Mechanical Stiffness

**Figure 5:**
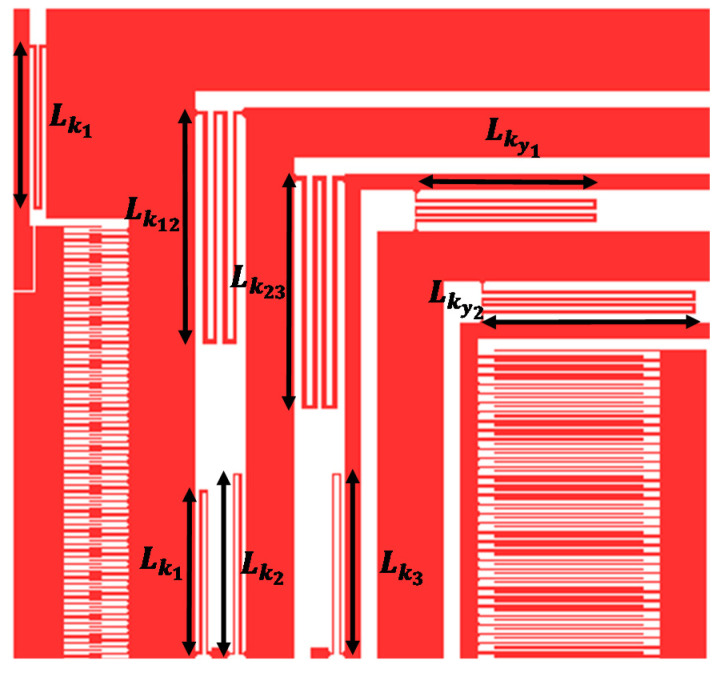
Mechanical suspension design for the proposed MEMS gyroscope design.

Figure 5 shows the configuration of mechanical springs attached to the masses. There is a total of seven sets of mechanical springs in the *x* and *y*-axis. Five sets of mechanical springs, namely $k_{1}$*,* $k_{2}$, $k_{3}$, $k_{12}$ and $k_{23}$ are placed such that they permit motion of the mass $m_{1}$, $m_{2}$ and $m_{f}$ in the drive direction, but restrict any motion in the sense direction. Another two sets of mechanical springs namely $k_{y_{1}}$ and $k_{y_{2}}$ are placed such that they allow movement of the mass $m_{3a}$ and $m_{3b}$ in the sensing direction only. This configuration of the mechanical springs allows the decoupling frame *m_f_* and the sense masses $m_{3a}$ and $m_{3b}$ to behave as a single mass in the drive direction.

Two types of springs, with different dimensions, are used in the MEMS gyroscope design i.e., double-folded and quadruple-folded serpentine springs. In the drive direction, the double-folded mechanical springs $k_{1}$_,_ with length $L_{k_{1}}$ and width $w_{k_{1}}$, connect the mass $m_{1}$ with the external anchor. Two double-folded springs $k_{2}$, with length and width given by $L_{k_{2}}$ and $w_{k_{2}}$, respectively, are used to connect the mass $m_{2}$ to the anchors. Two double-folded springs $k_{3}$, are used to connect the decoupling frame *m_f_* to the anchors and their lengths and widths are given by $L_{k_{3}}$ and $w_{k_{3}}$ respectively. Two mechanical springs, $k_{12}$ and $k_{23}$, are used to interconnect the drive masses, whereas $k_{y_{1}}$ and $k_{y_{2}}$ are used to interconnect the sense masses. A total of four mechanical springs $k_{12}$ with their length and width given by $L_{k_{12}}$ and $w_{k_{12}}$ respectively, are used to couple the masses $m_{1}$ and $m_{2}$. Four mechanical springs $k_{23}$ with length and width of $L_{k_{23}}$ and $w_{k_{23}}$ respectively, are used to connect the mass $m_{2}$ and decoupling frame $m_{f}$. In the sense direction, four springs $k_{y_{1}}$ are used to couple the frame $m_{f}$ with the sense mass $m_{3a}$. Their lengths and widths are given by $L_{k_{y_{1}}}$ and $w_{k_{y_{1}}}$ respectively. Another set of four springs $k_{y_{2}}$, is used to interconnect the masses $m_{3a}$ and $m_{3b}$, with length and width given by $L_{k_{y_{2}}}$ and $w_{k_{y_{2}}}$. The mechanical springs, both in drive and sense direction, can be modeled as fixed-guided beams and their equivalent mechanical stiffness can be written as:

(6) $$k_{j}=\frac{\beta }{\gamma }\left(\frac{12EI}{L_{k_{j}}^{3}}\right)=\frac{\beta }{\gamma }\frac{Etw_{k_{j}}^{3}}{L_{k_{j}}^{3}},$$

where *E* represents the Young’s Modulus of the material, *I* is the moment of inertia given by $I=\frac{tw^{3}}{12}$, *t* is the thickness, L is the length and w is the width of the beams. The subscript *j* represents the concerned mechanical spring and can have a value of 1, 2, 3, 12, 23, $y_{1}$ and $y_{2}$. The β and γ represent the number of folds in the serpentine springs and the total number of mechanical springs respectively.

### 4.3. Calculation of Differential Capacitance Change

For the proposed MEMS gyroscope design, an input angular rotation in the *z*-axis will result in the Coriolis force in the *y*-axis which will lead to the displacement in the 2-DoF sense mode oscillator. To detect the displacement in the sense mass, the sensing parallel plates are attached to the sense mass $m_{3b}$. These sensing plates are arranged in differential gap–antigap configuration and the overall capacitance change, corresponding to the sense mass displacement, can be calculated as [29]:

(7) $$\Delta C=\frac{2y\varepsilon_{o}l_{o}tN\left(d_{2}^{2}-d_{1}^{2}\right)}{\left(d_{2}^{2}-y^{2}\right)\left(d_{1}^{2}-y^{2}\right)},$$

where N is the number of parallel plates on each side,$\;\varepsilon_{o}$ is vacuum permittivity, $l_{o}$ is the overlap length of the fixed and moving parallel plates, y is the sense mass displacement and $d_{1}$ and $d_{2}$ are the small and large air gaps respectively.

### 4.4. Air Damping Analysis

For the proposed MEMS gyroscope, both the slide and squeezed film air damping contribute towards the energy dissipation. The slide film air damping is the only energy dissipation mechanism in the drive direction. The moving drive combs and moving sense parallel plates both move laterally in the drive direction and slide over the fixed parallel plates, hence contributing to the slide film air damping. The movement of masses in the sense direction results in the squeezed film air damping phenomenon. Due to the movement of sense mass, the moving parallel plates displace towards the fixed parallel plates and the thin air film between moving and fixed parallel plates is squeezed in. The slide film ($b_{sl}$) and squeezed film ($b_{sq}$) damping coefficients can be calculated as [30,31]:

(8) $$b_{sl}=N{µ}_{eff}A\left(\frac{1}{d_{1}}+\frac{1}{d_{2}}\right)$$

(9) $$b_{sq}=N_{s}{µ}_{eff}\;l_{o}t^{3}\left(\frac{1}{d_{1}^{3}}+\frac{1}{d_{2}^{3}}\right),$$

where N is the number of moving combs on each side of the mass, $N_{s}$ is the number of sensing parallel plates, A is the overlap area of the parallel plates, $l_{o}$ is the overlap length, ${µ}_{eff}$ is the effective viscosity of air and $d_{1}$ and $d_{2}$ are the smaller and larger gap sizes respectively. For drive combs, both $d_{1}$ and $d_{2}$ are of the same size i.e., *d* but for parallel sensing plates the values of $d_{1}$ and $d_{2}$ are 3 µm and 9 µm respectively.

The squeezed film air damping depends on the viscous, elastic and inertial effects of the thin air film present between the moving and fixed parallel plates. There are several types of flow regimes for the thin air film and are generally classified depending on a dimensionless value called the Knudsen number, $K_{n}$, which is defined as the ratio of the mean free path of air to the thickness of the air gap, and is given as:

(10) $$K_{n}=\frac{\lambda }{d_{1}},$$

where λ is the mean free path of air at a given operating temperature and pressure and $d_{1}$ is the nominal air gap thickness. The mean free path of the air can be calculated as [32]:

(11) $$\lambda =\frac{µ}{P}\;\sqrt{\frac{\pi K_{B}T}{2m_{air}}},$$

where µ is the air viscosity at atmospheric pressure, $m_{air}$ is the mass of air, P is the air pressure, T is the air temperature and $K_{B}$ is the Boltzman constant. The classification of flow regimes based on the $K_{n}$ values are discussed in [33]. For the proposed MEMS gyroscope, the smaller air gap between the sensing parallel plates is 3 µm which results in a $K_{n}$ value 0.0225 at atmospheric pressure and room temperature. This shows that, since this value is within 0.01 < $K_{n}$ < 0.1, the flow regime for the proposed MEMS gyroscope is slip flow. In the proposed MEMS gyroscope operating temperature range of −40 °C to 100 °C, the dominant flow regime remains slip flow with $K_{n}$ values of 0.0175 and 0.0275 at −40 °C and 100 °C, respectively.

The air damping force acting on the MEMS gyroscope can be either viscous, elastic or inertial and is dependent on the oscillation frequency of the mass. The relative effect of the air damping force is generally classified based on the dimensionless squeeze number *σ*, which is given as:

(12) $$\sigma =\frac{12{µ}_{eff}t^{2}\omega }{Pd_{1}^{2}},$$

where t is the characteristic length and for the proposed MEMS gyroscope represents the thickness of the parallel sensing plates, ${µ}_{eff}$ is the effective viscosity, *P* is the air pressure, $d_{1}$ is the smaller air gap and ω is operating frequency of the MEMS gyroscope. At low values of the squeeze number, the viscous damping force dominates the elastic force since the air between the fixed and moving parallel sense plates is not compressed completely. However, at high values of the squeeze number, the elastic damping force dominates, since the thin air film fails to escape from the gap between the fixed and moving parallel sensing plates. The term ${µ}_{eff}$ in Equation (12) is the effective viscosity of air at a given temperature and pressure, and is related to the air viscosity at ambient conditions, given as [34]:

(13) $${µ}_{eff}=\frac{µ}{1+9.638K_{n}^{1.1}}.$$

**Figure 6:**
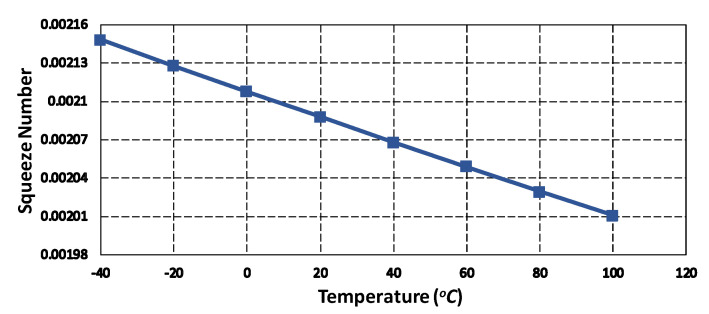
Effect temperature variations on the squeeze number for the proposed MEMS gyroscope.

The above equation shows that the ${µ}_{eff}$ is dependent on the $K_{n}$ and hence on the operating temperature. Figure 6 shows the effect of temperature variations on the squeeze number in the operating temperature range of −40 °C to 100 °C. The results show that the squeeze number decreases for the proposed MEMS gyroscope as the temperature increases. The inertial effect of damping force is generally explained by using Reynold’s number given as [35]:

(14) $$R_{e}=\frac{d_{1}^{2}\rho \omega }{{µ}_{eff}}.$$

where $d_{1}$ is the sense gap size, ρ is the density of air and ω is the operating frequency of the MEMS gyroscope. For low values of Reynolds number i.e., $R_{e}$ << 1, the inertial effects are very small and hence can be ignored. For the proposed MEMS gyroscope, the $R_{e}$ values are 0.00985 and 0.0105 at −40 °C to 100 °C. This shows that the inertial effect of the damping force is negligible for the MEMS gyroscope.

## 5. FEM Analysis for the MEMS Gyroscope

### 5.1. Modal Analysis

**Figure 7:**
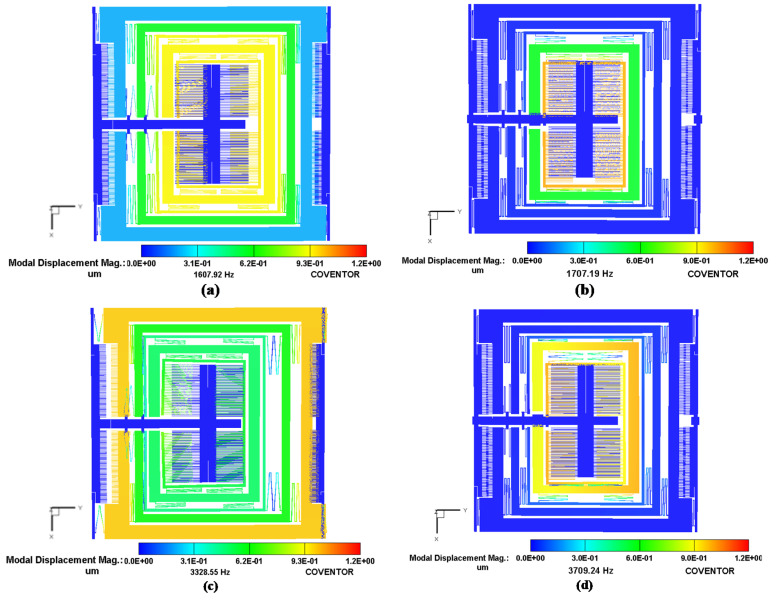
Modal analysis results and the corresponding mode shapes for MEMS gyroscope (**a**) 1st mode (1.607 kHz) (**b**) 2nd mode (1.707 kHz) and (**c**) 6th mode (3.329 kHz) and (**d**) 7th mode (3.709 kHz).

To determine the resonant frequency values and corresponding mode shapes for the MEMS gyroscope, a FEM-based analysis was carried out in the CoventorWare Analyzer module. Figure 7 shows the desired three drive mode shapes and two sense mode shapes for the MEMS gyroscope. The first, sixth and tenth modes were in the drive direction with a frequency of 1.608 kHz, 3.329 kHz and 4.822 kHz respectively. The second and seventh modes are in the sense direction with a frequency of 1.707 kHz and 3.709 kHz respectively. These results show that the two sense mode resonance frequencies are close to the first two drive mode frequencies, thus making the operational region of the MEMS gyroscope fall in the flat region between the resonance peaks. The third, fourth and fifth modes are out of the plane and twisting at 1.79 kHz, 1.9 kHz and 2 kHz respectively. These undesired modes separate from the flat region by at least 200 Hz. Table 3 compares the FEM-based modal analysis results with the analytical model, which show a close correspondence.

### 5.2. Pull-in Voltage Analysis

**Figure 8:**
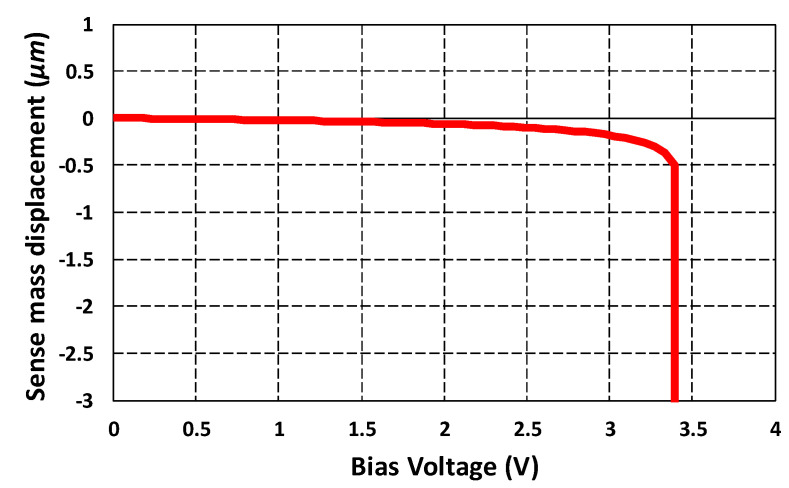
Sense mass displacement vs. applied bias voltage graph for the MEMS gyroscope.

The accurate operation of the parallel plate-based rotation sensing mechanism in the proposed gyroscope requires initial biasing of the sense plates. Due to the bias voltage, the moving sensing plate moves towards the fixed plate under the influence of an electrostatic force. The maximum value of the bias voltage is limited by the pull-in effect. The voltage at which the electrostatic force overcomes the mechanical force of the suspension beams is called the pull-in voltage and corresponding displacement is called the pull-in displacement. The pull-in voltage for the differential parallel sensing plates can be estimated as [36]:

(15) $$V_{pull-in}=\sqrt{\frac{8k_{y}d_{1}^{3}}{27l_{0}tN_{s}\varepsilon }},$$

where $k_{y}$ is the spring constant, $d_{1}$ is the initial air gap between the moving and fixed parallel plates, ε is the permittivity of free space and $N_{s}$ is the number of moving and fixed sensing plate pairs. To estimate the maximum bias voltage that can be applied to the sensing parallel plates in the proposed MEMS gyroscope, a pull-in voltage analysis was carried out in the CoventorWare MEMS+ module. Figure 8 shows the applied bias voltage versus sense mass displacement. The results show that pull-in occurs at a bias voltage of 3.4 V with a pull-in displacement of 0.5 µm. Thus, for the proposed MEMS gyroscope, the maximum bias voltage must be below 3.4 V.

### 5.3. Frequency Response Analysis

**Figure 9:**
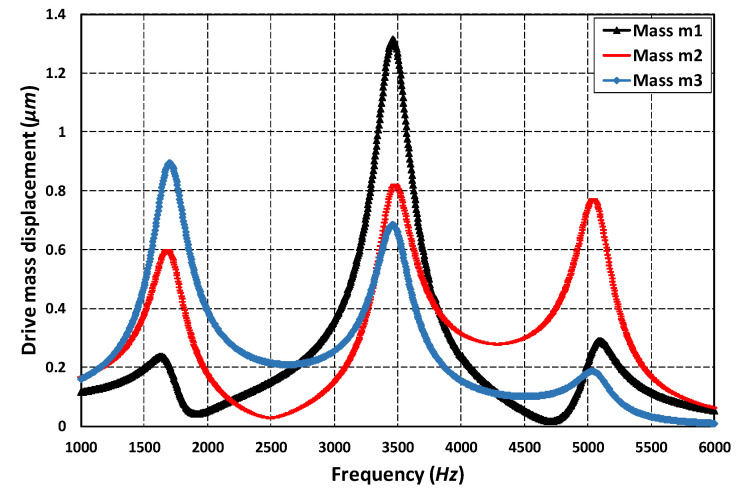
The frequency response of the 3-DoF drive mode oscillator.

To analyze the frequency response of the 3-DoF drive mode and 2-DoF sense mode oscillator, an FEM-based harmonic analysis is carried out in the CoventorWare analyzer module. A harmonic force was applied to the mass $m_{1}$ in the drive axis by applying an actuation voltage of 80 V DC and 5 V AC to the comb drive-based electrostatic actuators. The analysis was performed by considering the air damping effect. Figure 9 shows the frequency response of the 3-DoF drive mode oscillator. The results show that at the first resonant frequency, the displacement in the mass $m_{3}$ is dynamically amplified with an amplification ratio of three with respect to the mass $m_{1}$. The displacement in the mass $m_{1}$, $m_{2}$ and $m_{3}$ is 0.25 µm, 0.6 µm and 0.92 µm respectively. At the second and third resonant frequency, the displacement in the sense mass $m_{3}$ is minimum and reduced with respect to mass $m_{1}$. At the first resonant frequency, the three masses were in phase while at second and the third resonant frequency they were out of phase. In the flat operational region between the first and second resonant frequency, the displacement in the mass $m_{3}$ was dynamically amplified with an average amplification ratio of nearly two. The displacement amplitude of the mass $m_{3}$ was nearly 0.22 µm in the flat operational region.

**Figure 10:**
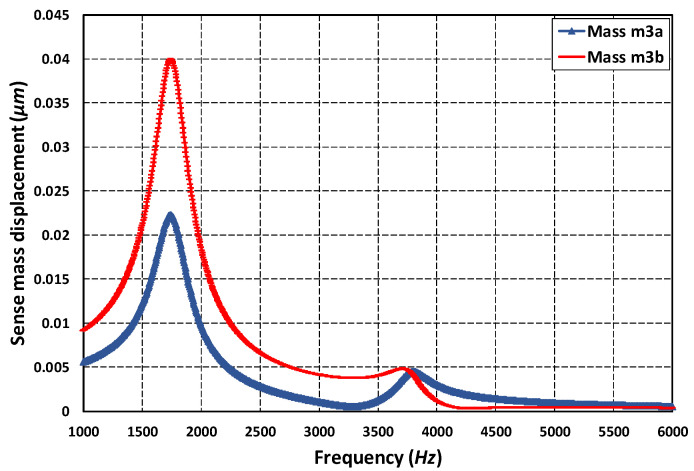
The frequency response of the 2-DoF sense mode oscillator.

Figure 10 shows the frequency response of the 2-DoF sense mode oscillator. At the first resonant frequency, the displacement in the mass $m_{3b}$ was dynamically amplified with an amplification ratio of 1.85. This is because both the mass $m_{3a}$ and $m_{3b}$ were in phase at the first sense mode resonant frequency. At the second resonant frequency, since both the masses were out of phase, thus, there was nearly zero dynamic amplification. However, in the flat operational region between the two resonant peaks, there was an amplified displacement in the mass $m_{3b}$ with respect to mass $m_{3a}$ with an average amplification ratio of nearly 2.6. The frequency response results shown in Figure 9 and Figure 10 show that the most suitable operational frequency region was between the resonant frequencies of 1.707 kHz and 3.329 kHz with a bandwidth of 1.622 kHz for the propose MEMS gyroscope design.

### 5.4. Oscillation Frequency-Dependent Air Damping Analysis

**Figure 11:**
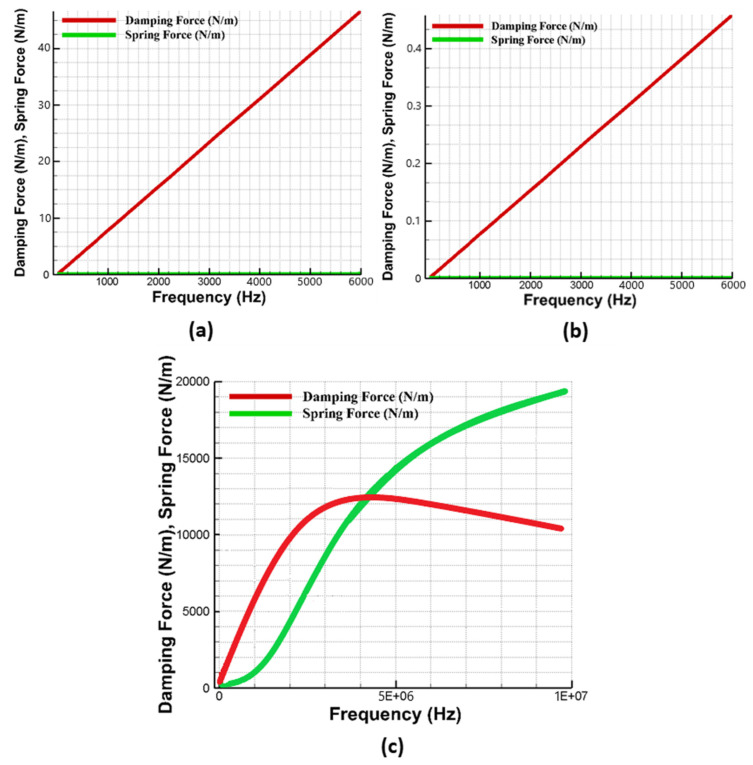
Effect of oscillation frequency on (**a**) squeezed film air damping for frequency up to 6 kHz (**b**) slide film air damping for frequency up to 6 kHz (**c**) squeezed film air damping for frequency up to 10 MHz.

The air damping force acting on the gyroscope can either be viscous, elastic or inertial depending on the oscillation frequency. To analyze the dominant air damping mechanism for the proposed MEMS gyroscope, a detailed FEM-based analysis was carried out in the DampingMM module of CoventorWare. Figure 11a,b shows the effect of oscillation frequency on the slide film and squeeze film air damping respectively at room temperature and pressure conditions and the relative contribution of both the viscous and elastic/spring damping forces. The results show that for the oscillation frequency in the range of 6 kHz, the viscous damping force was the main energy dissipation mechanism and effect of the elastic/spring force of air damping is negligible. These results were also verified by the Equation (12), since the squeeze number value for the MEMS gyroscope, at 6 kHz and room temperature and pressure was only 0.005. Figure 11c shows that with the higher values of oscillation frequency, the elastic/spring air forces became dominant and increased linearly with the oscillation frequency, while the viscous damping forces decreased. The FEM results presented in this section thus show that for the proposed MEMS gyroscope the main damping force will be viscous air damping. The values of squeezed film and slide film damping coefficients obtained through FEM analysis were $10.9\times 10^{-4}\;\mathrm{Ns}/m$ and $1.1\times 10^{-5}\;\mathrm{Ns}/m,$ respectively. The analytically-calculated values of both squeezed and slide film damping coefficients were $6.2\times 10^{-4}\;\mathrm{Ns}/m$ and $1.29\times 10^{-5}\;\mathrm{Ns}/m,$ respectively. The simulated value of the squeezed film damping coefficient was higher than the analytical value. This mismatch can be explained by the assumption that, in the analytical formulation, ideal sensing plate edge conditions were assumed while the simulation results from the DampingMM module of CoventorWare also accounted for the non-ideality of the sensing plate edges which leads to a more accurate and relatively higher value of damping coefficient [37].

### 5.5. Effect of Operating Temperature and Pressure Variations on MEMS Gyroscope

**Figure 12:**
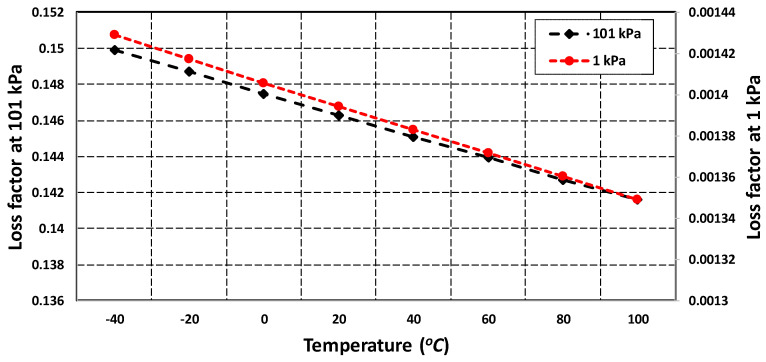
Energy loss factor for MEMS gyroscope with varying operating temperature and pressure values.

As discussed in Section 4.4, both the squeezed and slide film air damping are dependent on the effective air viscosity which is strongly influenced by the changes in the operating temperature and pressure conditions. The effect of temperature and pressure variations on the MEMS gyroscope was analyzed through FEM simulations in the DampingMM module of CoventorWare in the form of the energy loss function $\left(1/Q_{air}\right)$. Figure 12 shows that effect of change in temperature in the range of −40 °C to 100 °C on energy loss is negligible for the fixed values of atmospheric pressure at 1 kPa and 101 kPa. However, the energy loss function values were strongly dependent on the variation in the device operating pressure conditions. The results show that for a fixed temperature the value of energy loss function at 1 kPa was almost two orders of magnitude less than the value at 101 kPa.

**Figure 13:**
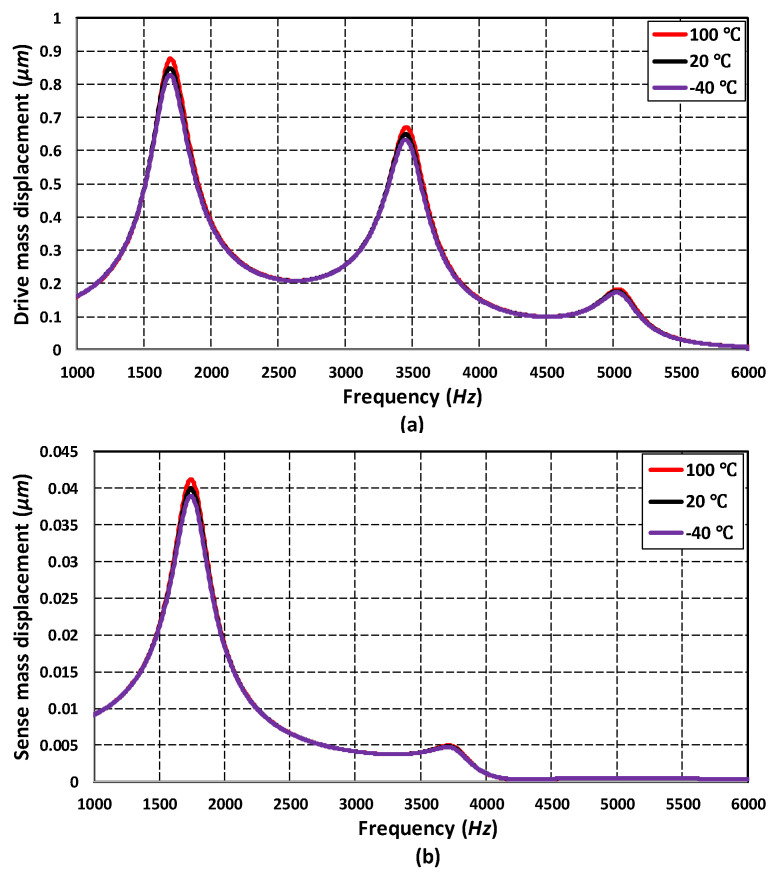
Effect of operating temperature variations on the frequency response of (**a**) drive mass $m_{3}$ and (**b**) sense mass $m_{3b}$.

**Figure 14:**
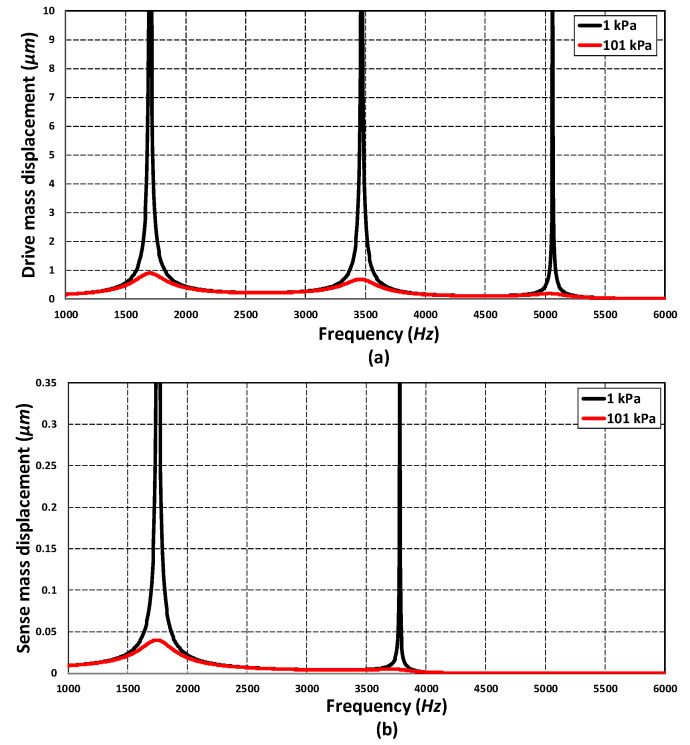
Effect of operating air pressure variations on the frequency response of (**a**) drive mass $m_{3}$ and (**b**) sense mass $m_{3b}$.

In addition to energy loss factor, the effect of varying temperature and pressure values on the frequency response of the absorber mass ($m_{3}$) in the drive mode and sense mass $m_{3b}$ in the sense mode was analyzed through FEM simulations. Figure 13a,b shows that the effect of temperature variations, in the range of −40 °C to 100 °C, was negligible on the response amplitude of the absorber mass both in the drive and sense direction. Figure 14a,b shows the effect of change in the operating air pressure, for 1 kPa and 101 kPa, on the frequency response of the absorber mass in the drive direction and sense mass in the sense direction. The results show that the displacement amplitude at resonance peaks was significantly increased by decreasing the operating air pressure. However, in the flat operational region between the resonant peaks, both in the drive and sense mode, there is a negligible change in the displacement amplitude of the masses. The results in Figure 13 and Figure 14 show that the proposed MEMS gyroscope is robust against both the operating temperature and air pressure variations in the flat operating region between the resonant peaks in the drive and sense mode.

### 5.6. Thermal Stability Analysis of MEMS Gyroscope

**Figure 15:**
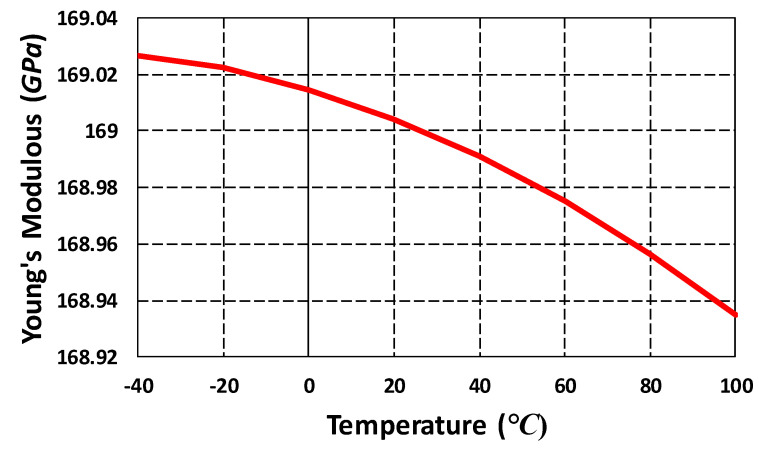
Young’s modulus of silicon in the temperature range of 40 °C to 100 °C.

The proposed MEMS gyroscope was designed considering the SOIMUMPs microfabrication process with silicon as a structural material. The material properties of silicon thin-film, including elasticity, are affected by the temperature. The change in Young’s modulus of silicon with temperature can result in variation in the resonant frequency of the MEMS gyroscope. The semi-empirical expression for the effect of temperature on Young’s modulus of the silicon has been reported in [38] and is given as:

(16) $$E\left(T\right)=E_{0}-BT\;exp\left(-\frac{T_{0}}{T}\right),$$

where $E_{0}$ is Young’s modulus at 0 K, *T* is the temperature, *B* and $T_{0}$ are constants which are dependent on the Grueneisen parameter, Debye temperature, Anderson–Grueneisen parameter and material volume at 0 K. For silicon, the values of *B* and $T_{0}$ are estimated as 15.8 MPa and 317 K respectively [39]. Figure 15 shows Young’s modulus value for the silicon for the operating temperature range of −40 °C to 100 °C for the proposed MEMS gyroscope. The results show that, for the desired operating temperature range for the MEMS gyroscope, the effect of temperature on stiffness variations is negligible.

**Figure 16:**
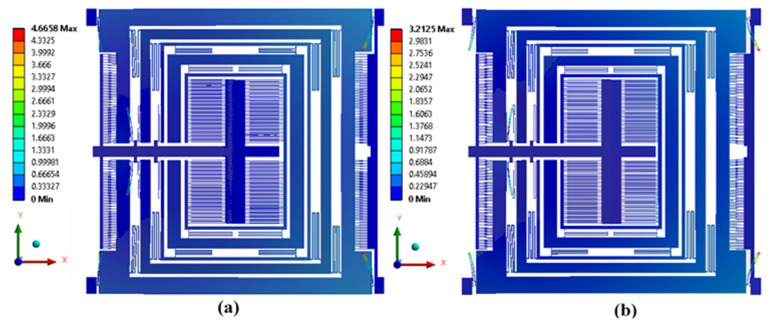
Structural thermal deformation in MEMS gyroscope (**a**) at 100 °C, (**b**) at −40 °C.

In addition to material properties changing, the device operating temperature may result in the thermal deformation in the microstructures. For the MEMS gyroscope, thermal deformation may result in the change in the gap and planarity between the electrostatic comb drive-based actuator and parallel plate-based capacitive sensor. To analyze the effect of the operating temperature in the range of −40 °C to 100 °C on the thermal deformation in the MEMS gyroscope, an FEM-based thermal analysis was carried out. The thermal deformation results in Figure 16a show that at 100 °C there was structural expansion in the MEMS gyroscope with a maximum value of 4.66 µm in the mechanical suspension beams. Figure 16b shows that at −40 °C, the mechanical structure contracted and maximum deformation in the mechanical springs was 3.21 µm. Although the deformation in the mechanical springs was high, the maximum expansion and contraction in the drive and sense mass was very low, with a maximum value of 0.4 µm at 100 °C and 0.27 µm at −40 °C. In the electrostatic comb drive actuator, attached to a mass $m_{1}$, the net effect of thermal deformation in the comb drives and hence on the electrostatic force is negligible due to symmetric deformation of the mass $m_{1}$.

**Figure 17:**
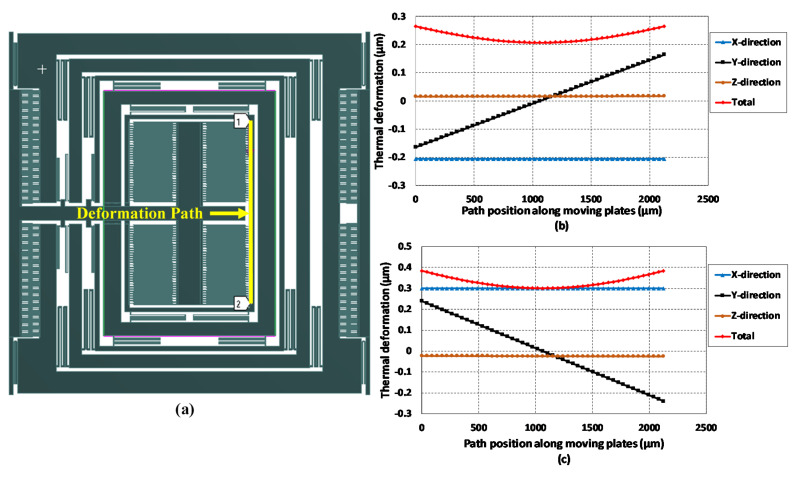
(**a**) Thermal deformation analysis path along with the sense mass $m_{3b}$ (**b**) thermal deformation at −40 °C. (**c**) thermal deformation at 100 °C.

To get more detailed information on the thermal deformation and its effect on the capacitive sensing plates, a deformation path is added in the FEM analysis along with the sense mass $m_{3b}$, as shown in Figure 17a. The results of the expansion and contraction along this path at −40 °C and 100 °C are shown in Figure 17b,c, respectively. The results show that the deformation due to temperature did not remain constant throughout the length of the sense mass and it was minimized at the center and increased on either side. The thermal deformation in the *x*, *y* and *z*-axis may lead to change in the initial overlap length between sensing parallel plates, initial gap size and overlap thickness values respectively. The maximum deformation in the *x*-axis was only 0.3 µm which was much less than the initial overlap length of 450 µm between parallel sensing plates. In the *z*-axis, thermal deformation was only 0.002 µm and was negligible to the initial overlap thickness value of 25 µm. However, in the sense *y*-axis, the deformation the mass $m_{3b}$ was 0.165 µm and 0.239 µm at −40 °C and 100 °C, respectively. This results in an effective initial air gap of nearly 2.84 µm and 2.76 µm between the parallel capacitive sensing plates. This decrease in the initial gap results in the decrease in the maximum pull-in voltage value and maximum input angular rate measurement.

### 5.7. Analysis of Fabrication Process Tolerances on Frequency Response

**Figure 18:**
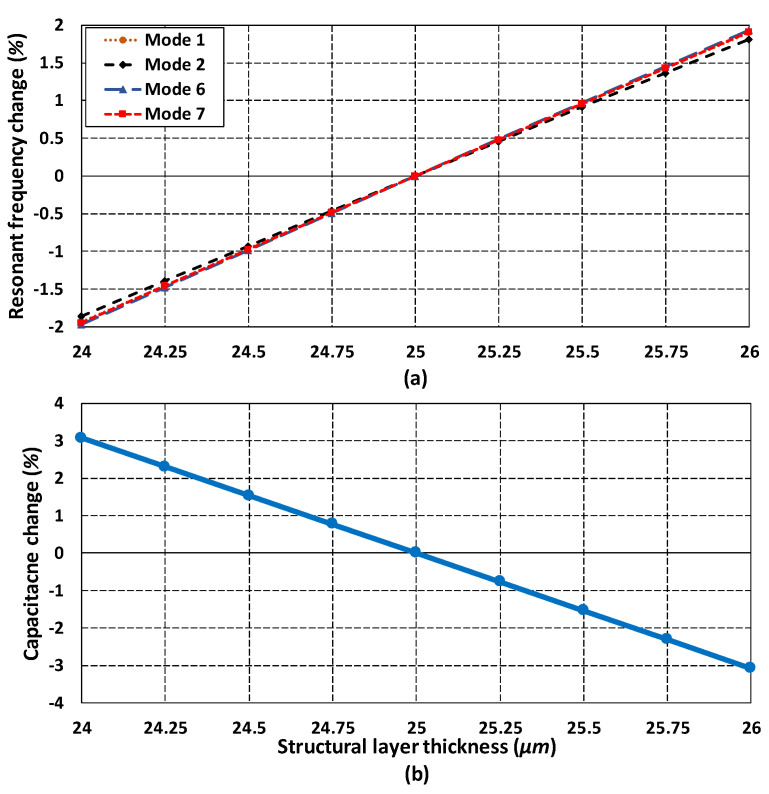
Effect of fabrication process tolerances on (**a**) resonant frequencies (**b**) capacitance change.

In addition to the environmental variations, the microfabrication process tolerances may also contribute towards uncertainty in the MEMS gyroscope performance. The SOIMUMPs is a relatively mature microfabrication process with tolerances of ±1 µm for the device layer and ±5 µm for the substrate layer. The main effect of device layer thickness is on the resonant frequency and sense capacitance change due to stiffness variation of mechanical springs and overlap area between the sensing parallel plates. The effect of change in structural layer thickness with 25 µm ± 1 µm was studied and its effect on the resonant frequency change and capacitance change was analyzed. Figure 18a shows that the effect of change in resonant frequency due to thickness tolerance of ±1 µm resulted in nearly 2% change in resonant frequency from the nominal values. Figure 18b shows that thickness tolerances result in a capacitance change variation of nearly 3.1% from the nominal values. These results show that the proposed MEMS gyroscope is robust against the microfabrication process tolerances.

## 6. Sensitivity Analysis of MEMS Gyroscope

### 6.1. Mechanical Sensitivity Analysis

**Figure 19:**
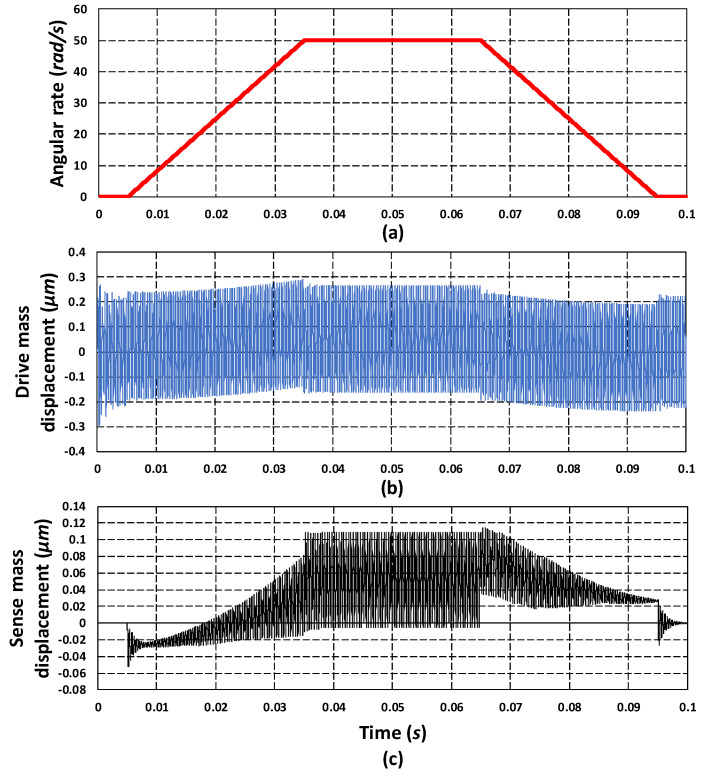
(**a**) Input angular velocity of 50 rad/s. (**b**) Oscillation response of the drive mass $m_{3}$. (**c**) Oscillation response of the sense mass $m_{3b}$.

As discussed in Section 2, the working of the proposed 3-DoF drive and 2-DoF sense mode MEMS gyroscope was based on the Coriolis effect. An applied actuation voltage to the comb drive-based electrostatic actuator results in the oscillation of the mass $m_{3}$ in the *x*-axis $\left(x_{3}\right)$. For an input rotation in the *z*-axis Ω, a Coriolis force $F_{c}=2m_{3a}\Omega {\dot{x}}_{3}$ was generated in the *y*-axis. This force results in a dynamically-amplified displacement $\left(y_{2}\right)$ in the sense mass $m_{3b}$. The proposed MEMS gyroscope was designed to be operated in the flat operating region between the first two resonant peaks of the drive and sense mode. To obtain the mechanical sensitivity for the MEMS gyroscope, transient analysis was carried out in the CoventorWare MEMS+ module. An actuation voltage of 80 V DC and 5 V AC was applied to the electrostatic actuator with a frequency of 2.5 kHz, which results in an oscillation amplitude of 0.22 µm in the mass $m_{3}$. For this oscillation amplitude and with a rotation of 50 rad/s in the *z*-axis, a Coriolis force of 1.943 µN was generated in the *y*-axis Figure 19a,b shows the input rotation and drive mass displacement, respectively. Figure 19c shows the *y*-axis displacement in the mass $m_{3b}$ corresponding to an induced Coriolis force with an amplitude of 0.114 µm. Assuming the linear relation between the input rotation and induced Coriolis force and hence sense mass displacement, the mechanical sensitivity for the proposed MEMS gyroscope was 0.00228 µm/rad/s. The analytical value of the oscillation amplitude of mass $m_{3}$ in the drive direction was 0.26 µm. The corresponding magnitude of the Coriolis force, in the *y*-axis for an input rotation of 50 rad/s was 2.2963 µN. The *y*-axis displacement in mass $m_{3b}$ due to this Coriolis force was 0.1347 µm and the mechanical sensitivity was 0.002694 µm/rad/s.

### 6.2. Integration of MEMS Gyroscope with Readout Electronics and Voltage Sensitivity Analysis

**Figure 20:**
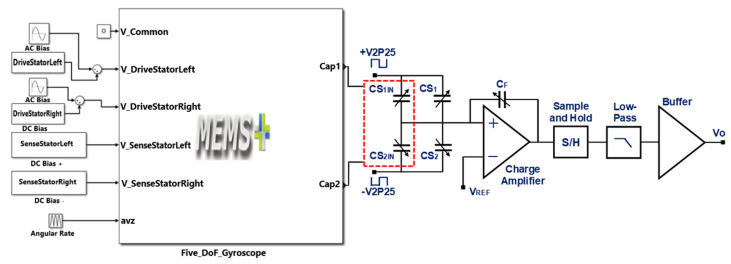
Integration of the MEMS gyroscope model with the readout electronics.

**Figure 21:**
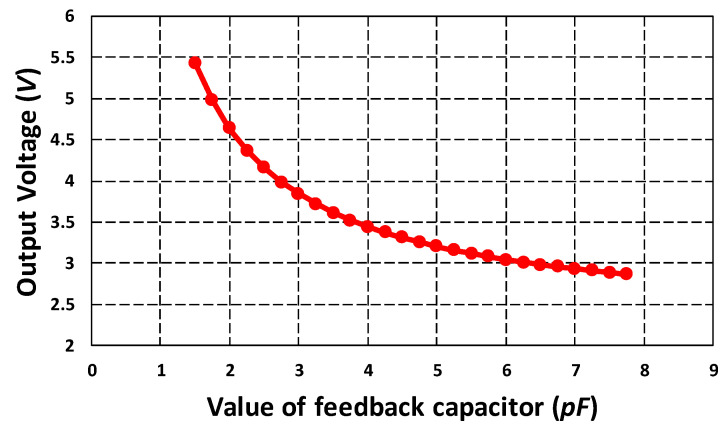
Effect of variation in the feedback capacitor on output voltage for MEMS gyroscope.

The voltage sensitivity of the MEMS gyroscope is not only dependent on the mechanical design, but also on the readout electronics used for the conversion of capacitance change, corresponding to an input rotation, to an equivalent output voltage. For the proposed MEMS gyroscope, a commercially available capacitance to voltage conversion Universal Readout IC^TM^ MS3110 is considered [40]. This IC is vastly used for signal conditioning of MEMS devices and provides an output voltage proportional to the change in differential capacitance. Figure 20 shows the integration of the proposed MEMS gyroscope with the internal circuitry of the MS 3110 IC in Matlab Simulink environment, which mentions the input and output ports of the MEMS gyroscope model, a combination of actuation voltages in the drive mode and a DC bias voltage applied to the sensing parallel plates. The differential output capacitances from the sensing parallel plates are shown as Cap1 and Cap2 and are connected as inputs (CS_1IN_ and CS_2IN_) to the MS 3110. The internal circuitry of the MS 3110 IC is shown as a simplified block diagram. The circuit contains a charge amplifier followed by a sample and hold circuit. A low pass filter is used for the signal conditioning and finally, the signal is passed through a buffer amplifier, which has offset trim functionality along with the output level selection. The MS 3110 IC has multiple programmable attributes, including the gain of the charge amplifier, the cut-off frequency of the filter and the buffer amplifier gain. The MS 3110 can operate in two modes with either a single-ended sense capacitor input or a differential input. In differential mode, the output capacitance from the MEMS gyro Cap1 and Cap2 may have mismatched values and may lead to a DC offset in the output values. In this case, the internal capacitors CS1 and CS2 of MS3110 are adjusted to reduce the DC offset by balancing the common mode capacitance. The output voltage of MS3110 IC can range between 0.5 V to 4 V and is given as:

(17) $$V_{o}=1.14\times G\times V2P25\times \;\frac{2\Delta C+\left(CS2-CS1\right)}{C_{F}}+V_{ref},$$

where G is the gain with a value of 2, V2P25 and $V_{ref}$ are set to 2.25 V, ΔC is the change in capacitance and $C_{F}$ is the feedback capacitor with a nominal value of 2.8 pF. The value of $C_{F}$ can be changed according to the sense capacitance range to make the output voltage reside within the desired range of 0.5 V to 4 V. Figure 21 shows the effect of variation in the $C_{F}$ on the output voltage of MS 3110 IC for the proposed MEMS gyroscope. The graph shows that for the MEMS gyroscope, for smaller values of $C_{F},$ the output voltage is higher than the upper limit of 4 V for the same input capacitance change. Moreover, if the capacitance change is too large then the output voltage may also increase beyond the maximum value of 4 V, so higher values of $C_{F}$ must be selected to keep the voltage in the acceptable range.

**Figure 22:**
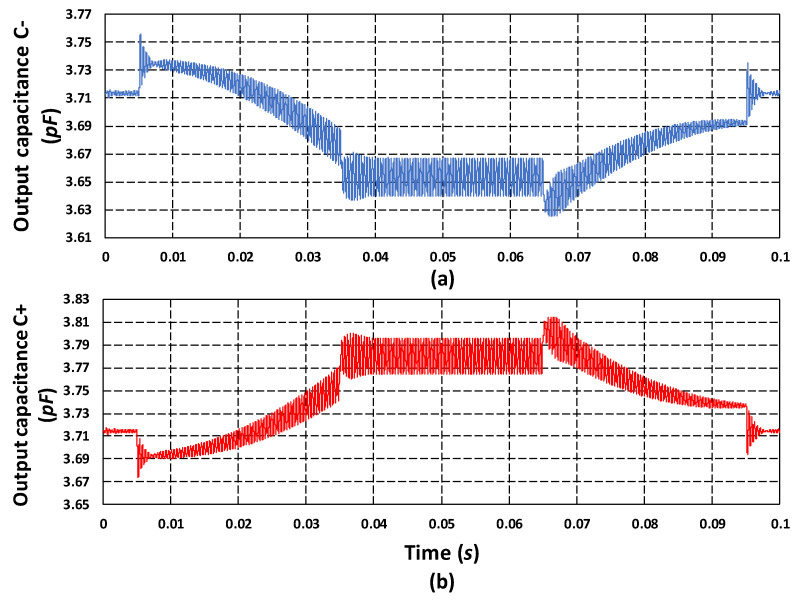
(**a**) Output sense capacitance C−. (**b**) Output sense capacitance C+.

Figure 22 shows the output capacitance response of the proposed MEMS gyroscope for an input rotation of 50 rad/s. The output capacitance C− in Figure 22a shows the decrease in the capacitance on one side of the differential parallel sensing plates of MEMS gyroscope with a value of 3.64 pF. Figure 22b shows the increase in capacitance C+ from rest value for on the other side of the parallel sensing plates, with a value of 3.795 pF. These results show that the net capacitance change (ΔC) for the proposed MEMS gyroscope is 155 fF for an input rotation of 50 rad/s which is in close agreement with an analytically calculated value of 136 fF using Equation (7). Assuming a linear relation between the net capacitance change, the capacitive sensitivity (ΔC/Ω) of the proposed MEMS gyroscope is 3.1 fF/rad/s and the corresponding output voltage of the MS3110 IC is 11.36 mV/rad/s.

## 7. Discussion

One of the most important performance parameters for MEMS devices is their reliability. The MEMS gyroscopes are supposed to work in harsh environments and under a wide range of temperature variations, usually in the range of −40 °C to 100 °C, while under constant oscillations. In addition to the structural design, the selection of material for the fabrication process of MEMS gyroscopes is very important, since reliability issues like fatigue limit under cyclic loading and stiffness degradation under the combined effect of loading and temperature (creep effect) are strongly dependent on the thin film material and its thickness. Most of the vibratory multi-DoF MEMS gyroscopes presented in the literature (given in Table 1) are fabricated either using silicon or Nickel thin films as a structural material. In comparison to Nickel, the silicon has much better mechanical properties and thermal stability. Thus, the selection of silicon as the structural material for our MEMS gyroscope gives us an added advantage of mechanical reliability and thermal stability. The complex structural design of multi-DoF vibratory MEMS gyroscopes, with multiple masses nested together, requires high accuracy during the microfabrication. Most of the silicon-based multi-DoF MEMS gyroscope designs presented in the literature are fabricated using in-house microfabrication processes. The costs involved in the development of such microfabrication facilities are very high. We believe that this is the reason that the research work on the development of non-resonant multi-DoF MEMS gyroscopes presented in literature is very limited.

The commercial foundry-based microfabrication of multi-DoF MEMS gyroscopes, based on the multiuser process concept, is a relatively low-cost solution for MEMS researchers. However, since the design rules offered by multiuser foundry services are fixed, the design must meet the fabrication process constraints and at the same time achieve the desired performance parameters. The multi-project wafer-based, low-cost and commercially available SOIMUMPs microfabrication process, offered by MEMSCAP Inc. USA, allows the use of silicon as a structural material with 25 µm thickness, which meets the basic requirements for MEMS gyroscope of high mass, mechanical reliability and thermal stability. The design rules of SOIMUMPs process for the design of multi-mass-based multi-DoF MEMS gyroscopes offer additional challenges, as discussed in Section 3. In this paper, we have focused on the design of multi-DoF MEMS gyroscope that not only meets the design rules of the fabrication process but also achieves better performance.

The performance characteristics of capacitive MEMS gyroscopes including voltage sensitivity, dynamic range and noise floor are dependent on the sense electronics parameters like resolution. The MS3110 has a resolution of 4 $\mathrm{aF}∕\sqrt{\mathrm{Hz}}$ and the minimum measurable angular rate for a MEMS gyroscope is dependent on this resolution. The resolution for the proposed MEMS gyroscope is estimated to be 0.0519 rad/s using the capacitive sensitivity and sense electronics resolution. The total noise equivalent in MEMS gyroscope is also dependent on the resolution of the sensing electronics. The total noise equivalent rate for the MEMS gyroscope at room temperature and atmospheric air pressure and with MS3110 readout electronics IC is 0.00328 $\mathrm{rad}/s/\sqrt{\mathrm{Hz}}$ with electrical noise equivalent rate and mechanical noise equivalent rate of 0.00129 $\mathrm{rad}/s/\sqrt{\mathrm{Hz}}$ and 0.00302 $\mathrm{rad}/s/\sqrt{\mathrm{Hz}}$ respectively. The dynamic range of the proposed MEMS gyroscope is estimated to be 69.2 dB. These values of noise equivalent and dynamic range for the proposed MEMS gyroscope are obtained by using the analytical expressions discussed in [41,42]. Table 4 shows the comparison of the proposed 3-DoF drive and 2-DoF sense mode gyroscope with the multi-DoF MEMS gyroscopes presented in the literature. In addition to the inherent advantages of being designed using a commercially available process, robustness against environmental variations and thermal stability the proposed MEMS gyroscope also exhibits better sensitivity and increased operational bandwidth in comparison to previous designs.

## 8. Conclusions

In this paper, we have presented a design of a 3-DoF drive mode and 2-DoF sense mode vibratory MEMS gyroscope considering the design rules of commercially available multi-user foundry process SOIMUMPs. The operational bandwidth of the proposed MEMS gyroscope is 1.62 kHz with a dynamic amplification ratio of 2 and 2.6 in the drive and sense direction respectively. The results showed that the effect of both squeezed and slide film air damping under varying operating temperature and pressure conditions is negligible in the operational bandwidth of gyroscope. The effect of temperature in the range of −40 °C to 100 °C on the stiffness variation of mechanical springs and hence on the dynamic response is also negligible for the MEMS gyroscope. The FEM-based structural thermal deformation analysis showed that the net effect of thermal deformation in the comb drives and hence on the electrostatic force is negligible due to symmetric deformation of the mass. In the sensing parallel plates, the thermal deformation is also symmetric and results in nearly 5% change in the initial air gap. The system modeling-based results showed that the proposed MEMS gyroscope has capacitive and voltage sensitivity of 3.1 fF/rad/s and 198.9 µV/(°/s) respectively when integrated with the commercially available capacitance to voltage conversion circuit. The dynamic range of the proposed MEMS gyroscope is estimated to be 69.2 dB with minimum and maximum angular rate of 0.0519 rad/s and 150 rad/s respectively. The total noise equivalent rate for the MEMS gyroscope considering sense electronics resolution is 0.00328 $\mathrm{rad}/s/\sqrt{\mathrm{Hz}}$.

## Figures and Tables

**Table 1:** Multi-degree of freedom (multi-DoF) non-resonant microelectromechanical systems (MEMS) gyroscopes presented in the literature.

References	Structural Layer		Configuration	Device Size	Sensitivity	Bandwidth
	Material	Thickness (µm)				
Acar et al. [13]	Polysilicon	2	2 DoF drive, 2 DoF sense	0.7 × 0.5 mm^2^	0.72 × 10^−3^ µm/°/s	23 Hz
Schofield et al. [14]	Silicon	75	1 DoF drive, 2 DoF sense	-	2.34 µV/(°/s)	600 Hz
Trusov et al. [15]	Silicon	50	1 DoF drive, 2 DoF sense	3 × 3 mm^2^	56 µV/(°/s)	250 Hz
Sahin et al. [16]	Silicon	100	1 DoF drive, 2 DoF sense		131 µV/(°/s)	1 kHz
Saqib et al. [18]	Nickel	20	3 DoF drive, 1 DoF sense	3.2 × 3 mm^2^	0.565 fF/rad/s, 0.052 × 10^−3^ µm/°/s	1.2 kHz
Saleem et al. [19]	Nickel	20	2 DoF drive, 1 DoF sense	2.4 × 1.6 mm^2^	0.045 fF/rad/s, 0.0105 × 10^−3^ µm/°/s	1.71 kHz
Verma et al. [20]	Nickel	9	1 DoF drive, 2 DoF sense	2 × 1.9 mm^2^	-	100 Hz
Acar et al. [21]	Polysilicon	100	1 DoF drive, 2 DoF sense	4 × 4 mm^2^	0.0308 mV/°/s	50 Hz
Riaz et al. [22]	Nickel	20	2 DoF drive, 1 DoF sense	2.2 × 2.6 mm^2^	-	1.4 kHz
Verma et al. [23]	Nickel	8	2 DoF drive, 1 DoF sense	2.13 × 1.93 mm^2^	-	1 kHz

**Table 2:** Design parameters of the proposed multi-DoF MEMS gyroscope.

Parameters	Values
Overall device size	4.2 mm × 4.2 mm
Structural layer thickness (t)	25 µm
Mass value of the mass $\left(m_{1}\right)$	2.5239 × 10^−7^ Kg
Mass value of the mass $\left(m_{2}\right)$	1.1203 × 10^−7^ Kg
Mass value of the decoupling frame $\left(m_{f}\right)$	3.234 × 10^−8^ Kg
Mass value of the mass $\left(m_{3a}\right)$	9.8125 × 10^−8^ Kg
Mass value of the mass $\left(m_{3b}\right)$	5.5343 × 10^−8^ Kg
Length of the parallel sensing plates (l)	500 µm
Width of the parallel sensing plates (w)	8 µm
Number of parallel sensing plates pair $\;\left(N_{s}\right)$	120
Overlap length between the moving and fixed parallel sensing plates $\left(l_{0}\right)$	450 µm
Smaller sense gap size $\left(d_{1}\right)$	3 µm
Larger sense gap size $\left(d_{2}\right)$	9 µm
Number of drive comb pairs $\left(N_{d}\right)$	240
Width of drive combs $\left(w_{d}\right)$	6 µm
Length of drive combs $\left(l_{d}\right)$	120 µm
Gap between drive combs (d)	3 µm

**Table 3:** Comparison of the analytical and FEM analysis-based modal frequency values.

Mode Shape	Analytical Model (kHz)	FEM Model (kHz)
1st (Drive mode)	1.738	1.608
2nd (Sense mode)	1.800	1.707
6th (Drive mode)	3.507	3.329
7th (Sense mode)	3.867	3.709
10th (Drive mode)	4.960	4.822

**Table 4:** Comparison of the proposed multi-DoF MEMS gyroscope design with the literature.

References	Structural Layer		Configuration	Device Size	Sensitivity	Bandwidth
	Material	Thickness (µm)				
Schofield et al. [14]	Silicon	75	1 Dof drive, 2 DoF sense	-	2.3 µV/(°/s)	600 Hz
Trusov et al. [15]	Silicon	50	1 Dof drive, 2 DoF sense	3 × 3 mm^2^	56 µV/(°/s)	250 Hz
Acar et al. [21]	Polysilicon	100	1 Dof drive, 2 DoF sense	4 × 4 mm^2^	0.0303 mV/(°/s)	50 Hz
Saqib et al. [18]	Nickle	20	3 Dof drive, 1 DoF sense	3.2 × 3 mm^2^	0.565 fF/rad/s	1.2 kHz
This work	Silicon	25	3 Dof drive, 2 DoF sense	4.2 × 4.2mm^2^	3.1 fF/rad/s, 198.9 µV/(°/s), 0.04 × 10^−3^ µm/(°/s)	1.62 kHz

## References

[1] Wu G.Q., Chua G.L., Gu Y.D. (2017). A dual-mass fully decoupled MEMS gyroscope with wide bandwidth and high linearity. Sens. Actuators A Phys..

[2] Shakoor R.I., Marc B., Iqbal S. (2018). Experimental evaluation of resonant frequencies with associated mode shapes and power analysis of thermally actuated vibratory microgyroscope. Microsyst. Technol..

[3] Yang J.S., Fang H.Y. (2003). A new ceramic tube piezoelectric gyroscope. Sens. Actuators A Phys..

[4] Dellea S., Giacci F., Longoni A.F., Langfelder G. (2015). In-plane and out-of-plane MEMS gyroscopes based on piezoresistive NEMS detection. J. Microelectromech. Syst..

[5] Bochobza-Degani O., Seter D.J., Socher E., Nemorivsky Y. (2000). A novel micromachined vibrating rate-gyroscope with optical sensing and electrostatic actuation. Sens. Actuators A Phys..

[6] Chuang W.C., Lee H.L., Chang P.Z., Hu Y.C. (2010). Review on the modeling of electrostatic MEMS. Sensors.

[7] Nguyen M.N., Ha N.S., Nguyen L.Q., Chu H.M., Vu H.N. (2017). Z-axis micromachined tuning fork gyroscope with low air damping. Micromachines.

[8] Xu Q., Hou Z., Kuang Y., Miao T., Ou F., Zhuo M., Xiao D., Wu X. (2019). A Tuning Fork Gyroscope with a Polygon-Shaped Vibration Beam. Micromachines.

[9] He C., Zhao Q., Huang Q., Liu D., Yang Z., Yan G. (2014). A MEMS vibratory gyroscope with real-time mode-matching and robust control for the sense mode. IEEE Sens. J..

[10] Balachandran G.K., Petkov V.P., Mayer T., Balslink T. (2015). A 3-axis gyroscope for electronic stability control with continuous self-test. IEEE J. Solid-State Circ..

[11] Jia J., Ding X., Gao Y., Li H. (2018). Automatic Frequency tuning technology for dual-mass MEMS gyroscope based on a quadrature modulation signal. Micromachines.

[12] Dyck C.W., Allen J.J., Huber R.J. (1999). Parallel-plate electrostatic dual-mass resonator. Micromachined Devices and Components V.

[13] Acar C., Shkel A.M. (2003). Nonresonant micromachined gyroscopes with structural mode-decoupling. IEEE Sens. J..

[14] Schofield A.R., Trusov A.A., Shkel A.M. (2008). Effects of operational frequency scaling in multi-degree of freedom MEMS gyroscopes. IEEE Sens. J..

[15] Trusov A.A., Schofield A.R., Shkel A.M. (2009). Performance characterization of a new temperature-robust gain-bandwidth improved MEMS gyroscope operated in air. Sens. Actuators A Phys..

[16] Sahin K., Sahin E., Alper S.E., Akin T. (2009). A wide-bandwidth and high-sensitivity robust microgyroscope. J. Micromech. Microeng..

[17] Erismis M. (2013). Design and modeling of a new robust multi-mass coupled-resonator family with dynamic motion amplification. Microsyst. Technol..

[18] Saqib M., Mubasher Saleem M., Mazhar N., Awan S.U., Shahbaz Khan U. (2018). Design and analysis of a high-gain and robust multi-DOF electro-thermally actuated MEMS gyroscope. Micromachines.

[19] Saleem M.M., Bazaz S.A. (2011). Design and robustness analysis of structurally decoupled 3-DoF MEMS gyroscope in the presence of worst-case process tolerances. Microsyst. Technol..

[20] Verma P., Khan K.Z., Khonina S.N., Kazanskiy N.L., Gopal R. (2016). Ultraviolet-LIGA-based fabrication and characterization of a nonresonant drive-mode vibratory gyro/accelerometer. J. Micro-Nanolithogr. MEMS MOEMS.

[21] Acar C., Shkel A.M. (2006). Inherently robust micromachined gyroscopes with 2-DOF sense-mode oscillator. J. Microelectromech. Syst..

[22] Riaz K., Bazaz S.A., Saleem M.M., Shakoor R.I. (2011). Design, damping estimation and experimental characterization of decoupled 3-DoF robust MEMS gyroscope. Sens. Actuators A Phys..

[23] Verma P., Shekhar C., Arya S.K., Gopal R. (2015). New design architecture of a 3-DOF vibratory gyroscope with robust drive operation mode and implementation. Microsyst. Technol..

[24] Zhu X.H., Chu H.J., Shi Q., Qiu A.P., Su Y. (2008). Experimental study of compensation for the effect of temperature on a silicon micromachined gyroscope. Proc. Inst. Mech. Eng. Part N J. Nanoeng. Nanosyst..

[25] Guo Z., Fu P., Liu D., Huang M. (2018). Design and FEM simulation for a novel resonant silicon MEMS gyroscope with temperature compensation function. Microsyst. Technol..

[26] Cui M., Huang Y., Wang W., Cao H. (2019). MEMS Gyroscope Temperature Compensation Based on Drive Mode Vibration Characteristic Control. Micromachines.

[27] Dwivedi A., Khanna G. (2019). Numerical simulation and modelling of a novel MEMS capacitive accelerometer based microphone for fully implantable hearing aid. Microsyst. Technol..

[28] Cowen A., Hames G., Monk D., Wilcenski S., Hardy B. SOIMUMPs Design Handbook (Revision 8.0.). http://www.memscap.com.

[29] Edalatfar F., Yaghootkar B., Qureshi A.Q.A., Azimi S., Bahreyni B. (2016). Design, fabrication and characterization of a high performance MEMS accelerometer. IEEE Sens. J..

[30] Bao M., Yang H. (2007). Squeeze film air damping in MEMS. Sens. Actuators A Phys..

[31] Abdolvand R., Amini B.V., Ayazi F. (2007). Sub-micro-gravity in-plane accelerometers with reduced capacitive gaps and extra seismic mass. J. Microelectromech. Syst..

[32] Mol L., Rocha L.A., Cretu E., Wolffenbuttel R.F. (2009). Squeezed film damping measurements on a parallel-plate MEMS in the free molecule regime. J. Micromech. Microeng..

[33] Syed W.U., Brimmo A., Waheed O., Bojesomo A., Ali M.H., Ocak I., Elfadel I.A.M. (2017). Numerical modeling and validation of squeezed-film damping in vacuum-packaged industrial MEMS. J. Micromech. Microeng..

[34] Veijola T., Raback P. (2007). Methods for solving gas damping problems in perforated microstructures using a 2D finite-element solver. Sensors.

[35] Morris C.J., Forster F.K. (2004). Oscillatory flow in microchannels. Exp. Fluids.

[36] Somà A., Saleem M.M., De Pasquale G. (2016). Effect of creep in RF MEMS static and dynamic behavior. Microsyst. Technol..

[37] (2018). CoventorWare Analyzer Field Solver Reference.

[38] Wachtman J.B., Tefft W.E., Lam D.G., Apstein C.S. (1961). Exponential temperature dependence of Young’s modulus for several oxides. Phys. Rev..

[39] Gysin U., Rast S., Ruff P., Meyer E., Lee D.W., Vettiger P., Gerber C. (2004). Temperature dependence of the force sensitivity of silicon cantilevers. Phys. Rev. B.

[40] Datasheet MUCRI (2004). MS3110 Universal Capacitive ReadoutTM IC.

[41] Bao M.H. (2000). Micro Mechanical Transducers: Pressure Sensors, Accelerometers and Gyroscopes.

[42] Apostolyuk V. (2006). Theory and Design of Micromechanical Vibratory Gyroscopes in MEMS/NEMS.

